# Provision of HIV viral load testing services in Zimbabwe: Secondary data analyses using data from health facilities using the electronic Patient Monitoring System

**DOI:** 10.1371/journal.pone.0245720

**Published:** 2021-01-22

**Authors:** Tsitsi Apollo, Kudakwashe C. Takarinda, Andrew Phillips, Chiratidzo Ndhlovu, Frances M. Cowan

**Affiliations:** 1 Department of HIV and TB, Ministry of Health and Child Care, Harare, Zimbabwe; 2 Department of Medicine, University of Zimbabwe College of Health Sciences, Harare, Zimbabwe; 3 Centre for Operations Research, International Union Against TB and Lung Disease, Paris, France; 4 Institute for Global Health, UCL, London, United Kingdom; 5 Centre for Sexual Health and HIV/AIDS Research (CeSHHAR), Harare, Zimbabwe; 6 Liverpool School of Tropical Medicine, Liverpool, United Kingdom; University of the Witwatersrand, SOUTH AFRICA

## Abstract

**Introduction:**

Routine viral load (VL) testing among persons living with Human Immunodeficiency Virus (PLHIV) enables earlier detection of sub-optimal antiretroviral therapy (ART) adherence and for appropriate management of treatment failure. Since adoption of this policy by Zimbabwe in 2016, the extent of implementation is unclear. Therefore we set out to determine among PLHIV ever enrolled on ART from 2004–2017 and in ART care for ≥12 months at health facilities providing ART in Zimbabwe: numbers (proportions) with VL testing uptake, VL suppression and subsequently switched to 2^nd^-line ART following confirmed virologic failure.

**Materials and methods:**

We used retrospective data from the electronic Patient Monitoring System (ePMS) in which PLHIV on ART are registered at 525 public and 4 private health facilities.

**Results:**

Among the 392,832 PLHIV in ART care for ≥12 months, 99,721 (25.4%) had an initial VL test done and results available of whom 81,932 (82%) were virally suppressed. Among those with a VL>1000 copies/mL; 6,689 (37.2%) had a follow-up VL test and 4,086 (61%) had unsuppressed VLs of whom only 1,749 (42.8%) were switched to 2^nd^-line ART. Lower age particularly adolescents (10–19 years) were more likely (ARR 1.34; 95%CI: 1.25–1.44) to have virologic failure.

**Conclusion:**

The study findings provide insights to implementation gaps including limitations in VL testing; low identification of high- risk PLHIV in care and lack of prompt utilization of test results. The use of electronic patient-level data has demonstrated its usefulness in assessing the performance of the national VL testing program. By end of 2017 implementation of VL testing was sub-optimal, and virological failure was relatively common, particularly among adolescents. Of concern is evidence of failure to act on VL test results that were received. A quality improvement initiative has been planned in response to these findings and its effect on patient management will be monitored.

## Introduction

Over the last decade significant progress has been made in scaling up human immunodeficiency virus (HIV) treatment programs in low to middle-income countries (LMICs), with over 21 million people globally receiving antiretroviral therapy (ART) by 2017 [1].

Viral load (VL) is a direct measure of HIV treatment response. Studies have shown that when an ART regimen is fully effective (i.e. no drug resistance) and adherence is optimized, most PLHIV become virally suppressed with undetectable VLs (<50–1000 copies/ mL) within six months of ART initiation [2, 3].

The World Health Organization recommends VL testing for monitoring adherence to, and effectiveness of, ART among persons with HIV (PLHIV) to enable earlier detection of poor adherence and management of treatment failure. Viral suppression allows immune recovery and renders HIV- infected individuals non-infectious [4]. Virologic monitoring is universal in high-income countries; however, it is limited in some low-income countries.

By the end of 2017, Zimbabwe had a population of 13 million people and an estimated 1.3 million adults and children living with HIV of whom approximately 1.1 million patients were receiving ART [5]. The Zimbabwe National ART programme guidelines recommend use of VL testing to monitor HIV treatment response, with testing performed at 6 months, 12 months and then yearly following ART initiation [6]. In Zimbabwe, about 73% of the PLHIV reported knowing their HIV status, and 86.6% of those who knew their status report being on ART [7]. It is estimated that 15% of those on ART had a VL ≥ 1,000 copies/ml and therefore they had not achieved VL suppression. By December 2017, fewer than 30% of PLHIV in ART care had received at least one VL test in the public sector (estimates based on VL tests performed, compared with a national target of 70% by 2017) [8]. Due to resource constraints, prior to 2016 sub-populations were prioritized and these included pregnant women, children and PLHIV suspected of treatment failure based on immunological or clinical criteria and among PLHIV who had disengaged from care. Nevertheless, anecdotal reports suggest variability across health facilities with missed opportunities for effective monitoring and management of PLHIV [9, 10]. As per national ARV Guidelines; PLHIV who have an unsuppressed VL (defined as >1,000 copies/mL) undergo enhanced adherence counselling (EAC), followed by a repeat VL test after 3 months with a switch in ART regimen (to second or third line therapy as appropriate) if the VL remains unsuppressed [6]. However, the extent to which these guidelines were followed under routine program conditions was unclear.

Our specific study objectives were to determine among PLHIV initiated on ART in Zimbabwe between 01 August 2004 and 01 January 2017: i) the number (proportion) who received VL testing, and were virally suppressed ii) among those with an initial unsuppressed VL, the number (proportion) with a subsequent VL test following EAC sessions, iii) the number (proportion) switched to 2^nd^ line ART among those with confirmed virological failure iv) and their associated demographic and clinical factors.

## Materials and methods

### Study design

This was a retrospective cohort study design using routinely collected programme data.

### Setting

Zimbabwe offers HIV treatment and care services free of charge in public health facilities and selected not-for-profit private health facilities, and these are integrated with provision of general health services. There are 1,620 public health facilities offering HIV treatment and care services in Zimbabwe across 10 provinces (including Harare and Bulawayo metropolitan provinces). These health facilities are stratified into a four-tiers consisting of rural/urban primary health care (PHC) facilities at the lowest level, district hospitals and selected faith-based hospitals at the secondary referral level, 8 provincial hospitals at the third referral level and 5 central hospitals at the fourth referral level. Health services are provided by nurses at the PHCs with doctors only found from secondary referral level and above were theatre and laboratory facilities are available.

### Study sample

Our study focused on data from 529 health facilities (525 public and 4 private not-for-profit health facilities) providing HIV treatment and care services and using the national Electronic Patient Monitoring System (ePMS). The ePMS is an electronic-last system for capturing patient-level demographic, clinical, and laboratory information at enrolment and for follow-up review visits among PLHIV registered in HIV care. The system is in use at 529 high volume health facilities (with ≥500 PLHIV on ART by December 2012) out of 1,620 health facilities providing ART services in Zimbabwe. Despite the ePMS coming into use from 2012 onwards, historical patient-visit data were entered for patients who were alive in ART care and had been enrolled into care dating back to 2004 when ART was first offered in the public sector. To ensure that analysis was restricted to those who were eligible for VL testing, we included in our study only PLHIV enrolled into ART care between 01 August 2004 and 01 January 2017 (regardless of age) who were still alive in ART care for at least 12 months beyond 30 June 2017 at health facilities with the ePMS.

### HIV diagnosis and treatment services in Zimbabwe

Most PLHIV are HIV diagnosed at health facilities through provider-initiated or voluntary HIV testing. Alternatively, HIV testing is conducted in the community through outreach programmes and index testing. The HIV testing algorithm is based on the WHO 2015 HIV testing algorithm where an initial test is done using an Alere Determine HIV-1/2 Ag/Ab Combo rapid HIV test or Standard Q rapid HIV test followed by a confirmatory rapid HIV test using a First Response HIV 1-2-0 Card test or Chembio if the initial test is HIV-positive. If these tests are discordant they are repeated in parallel, with a final result concluded by concordant results. If still discordant, a third test (INSTI) is performed. Once diagnosed positive for HIV, a patient is retested for verification before enrolment into HIV care where they are prepared and counselled for ART [6].

By end of 2018, the preferred first-line ART regimen among adolescents (10–19 years) and adults (20+ years) was a once-daily fixed-dose combination of Tenofovir (TDF) 300mg plus Lamivudine (3TC) 300mg plus Efavirenz (EFV) 400mg. The alternative first-line regimen was a combination of TDF (300mg)+3TC (300mg) and Nevirapine (NVP) 200mg. However, use of Nevirapine for HIV treatment has since been phased out. Zidovudine could be used as a substitute for Tenofovir. For those with confirmed virologic failure, the preferred second-line ART regimen consisted of Zidovudine (AZT) + 3TC + Atazanavir (ATV/r) or Lopinavir (LPV/r) if TDF was used in the first-line regimen. Alternatively, TDF could be used if AZT was used in the first-line ART regimen. Those failing second-line ART were referred for specialist assessment which included a viral load test and genotype testing prior to commencing third line ART. Medicines used as 3^rd^ line ART consist of Dolutegravir (DTG) 50mg and Darunavir (600mg)/Ritonavir (100mg) twice daily. Raltegravir (400mg) twice daily could be used when DTG was not available [6].

### Viral load testing services in Zimbabwe

**Fig 1:**
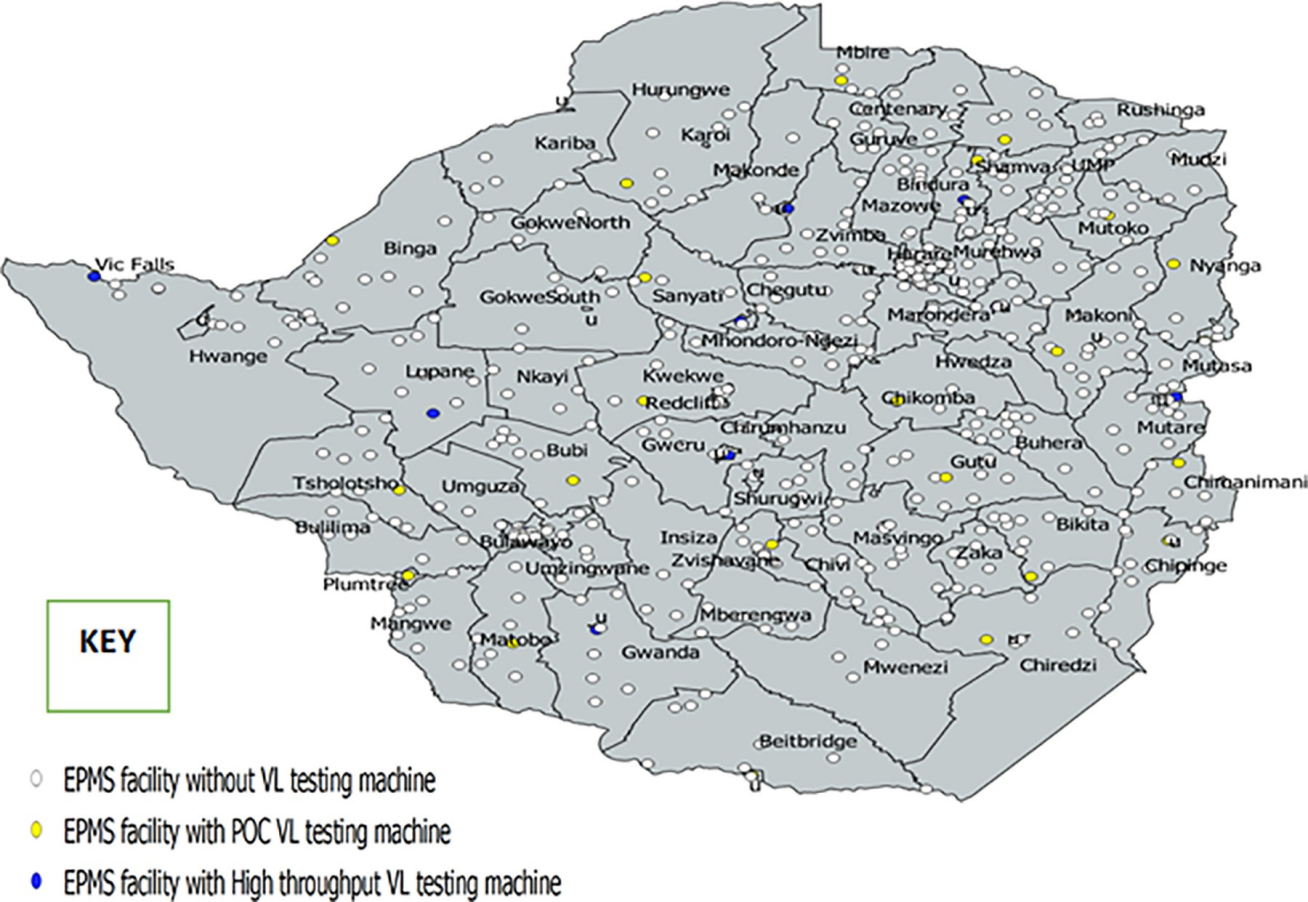
Distribution of health facilities using the ePMS versus those with highthroughput viral load testing machines and point-of-care testing machines. ePMS = electronic Patient Monitoring System; POC VL = point-of-care viral load.

In Zimbabwe, VL testing is offered free of charge at public health facilities and has been decentralized to six provinces with 18 high through-put platforms situated in laboratories at selected third- and fourth- level referral health facilities. VL samples are collected from health facilities using either Dried Blood Spot (DBS) samples for testing using Biomérieux NucliSENS and Abbott m2000 platforms or whole blood samples for testing using the Roche Taqman as well as the Abbott m2000 platforms. These samples are then transported to collection points (hubs) prior to being couriered to the laboratory. There are also 25 second-level health facilities with SAMBA Semi-Q point-of-care VL machines although they have very low testing capacity. **Fig 1** shows health facilities with the ePMS versus those with VL machines onsite.

CD4 testing still remains important for assessing patients with CD4<100 cells/mL requiring routine screening for crypotococcal meningitis using an antigen test and TB screening using the urine lipoaribomannan assay test. The CD4 test was also important for determining ART eligibility among those with CD4 cells counts below the 200-, 350- and eventually 500- thresholds overtime, prior to adoption of the “HIV treat-all” approach in mid-2016 where all HIV-diagnosed PLHIV were eligible for ART initiation, regardless of CD4 count.

### Data management

The primary source document for data variables included in this study was the individual ART care booklet which is entered in the ePMS. Data variables abstracted from the EPMS for purposes of this study include sex, date of birth, date of ART initiation, WHO clinical staging, CD4 cell count, current pregnancy status, anemia status at enrolment, dates of VL sample collection and VL test results and date of switching to 2^nd^-line ART. Current pregnancy status referred to the last visit when data was collected from 30 June 2017 onwards among the women living with HIV (WLHIV) who were included in this study. This definition was chosen since women are eligible for VL testing during pregnancy in order to assess whether they are at high risk or low risk of mother-to-child transmission of HIV. Anemia was defined as having hemoglobin levels <13.5g/dL among men or <12g/dL among women.

The ePMS is an offline system with a MySQL database format (Oracle International Corporation, Redwood City, CA, USA) with a Microsoft Access front-end. Upon receipt of routine quarterly submissions of health facility-level data back-up files for the October-December 4 2018 period, selected variables were imported using Stata version 15 (StataCorp, College Station, Texas USA) through the “OBDC data source” command into separate datasets for demographics, clinical data, laboratory tests and patient follow-up visit data. These were separate datasets which were merged into health facility-specific datasets and then appended into one large dataset.

The ART number (which is a unique identifier assigned to a patient upon enrolment into life-long HIV care) was used to de-duplicate data for patients who transferred from one health facility to another with the ePMS installed and also to account for their current ART outcomes, especially for patients who were self-transfers and might have been misclassified as lost-to-follow up. However, this was not possible for a patient who might have self-transferred to another health facility and presented as a new patient and therefore assigned a new ART number. We also could not determine the true ART outcomes among self-transfers who might have relocated to other health facilities without the ePMS, given that this is available in approximately 40% (634/1,560) of all health facilities.

### Statistical analysis

Categorical variables were summarized by frequencies and proportions whilst continuous skewed variables were summarized as medians and interquartile ranges.

The outcomes of interest were the proportion of those on ART who i) had ever had a VL test ii) had an initial unsuppressed VL iii) had a repeat VL test (among those with an initial unsuppressed VL), iv) had confirmed virologic failure and v) had switched to 2^nd^-line ART following confirmed virologic failure. An unsuppressed viral load is any VL measurement that is ≥1,000 copies/ml whilst a confirmed virologic failure is defined as a VL ≥1,000 copies/ml based on two consecutive VL measurements within a 3-month interval following enhanced adherence counselling and after being on ART for at least 6 months according to WHO [3].

However in this study, a confirmed virologic failure was defined as a documented follow-up unsuppressed VL received within 9 months after date of the initial VL result and after being on ART for at least 6 months. This was assuming that 3 months after the initial unsuppressed VL there would be EAC sessions whilst the 3 to 6 months after EAC was to account for date of receipt of the VL result. The censoring date for this study was therefore set at 31 December 2018 to account for those initiated on ART by 01 January 2017.

Bivariate analyses using the Chi square test were done to determine associations between various demographic and clinical characteristics and ever having a VL test done and to compare proportions with VL suppression by gender between the different age groups. We also calculated unadjusted and multivariable-adjusted relative risks and their 95% confidence intervals in order to determine factors associated with having a follow-up unsuppressed VL using a generalized linear model with a log link and poisson distribution with a robust error variance. All variables with a p value<0.25 in the Chi Square test for associations with having an unsuppressed follow-up VL result were included in the multivariate adjusted regression model. Variables adjusted for were province, sex, age group, current pregnancy status, WHO clinical staging, baseline CD4 cell count, ART regimen prior to a first VL test, time to the first VL test since ART initiation and level of health care facility. All p-values less than 0.05 were statistically significant.

### Ethics approval

This study was approved by the Medical Research Council of Zimbabwe (Approval Number: MRCZ/A/2160) and the Joint Research Ethics Committee (JREC) for University of Zimbabwe College of Health Sciences and Parirenyatwa Group Hospitals (Approval Number: JREC/12/17). The ethics committee waived the requirement for informed consent by participants as VL testing is part of ministry of health routine standard of care for monitoring HIV treatment response. Data were also fully anonymized as patient names were excluded in the abstraction of data. Instead patient ART numbers (which are unique identifiers assigned to patients upon enrolment in HIV care) were abstracted and later replaced in the electronic database with a sequential number to ensure patients cannot be traced back to the facility.

## Results

**Fig 2:**
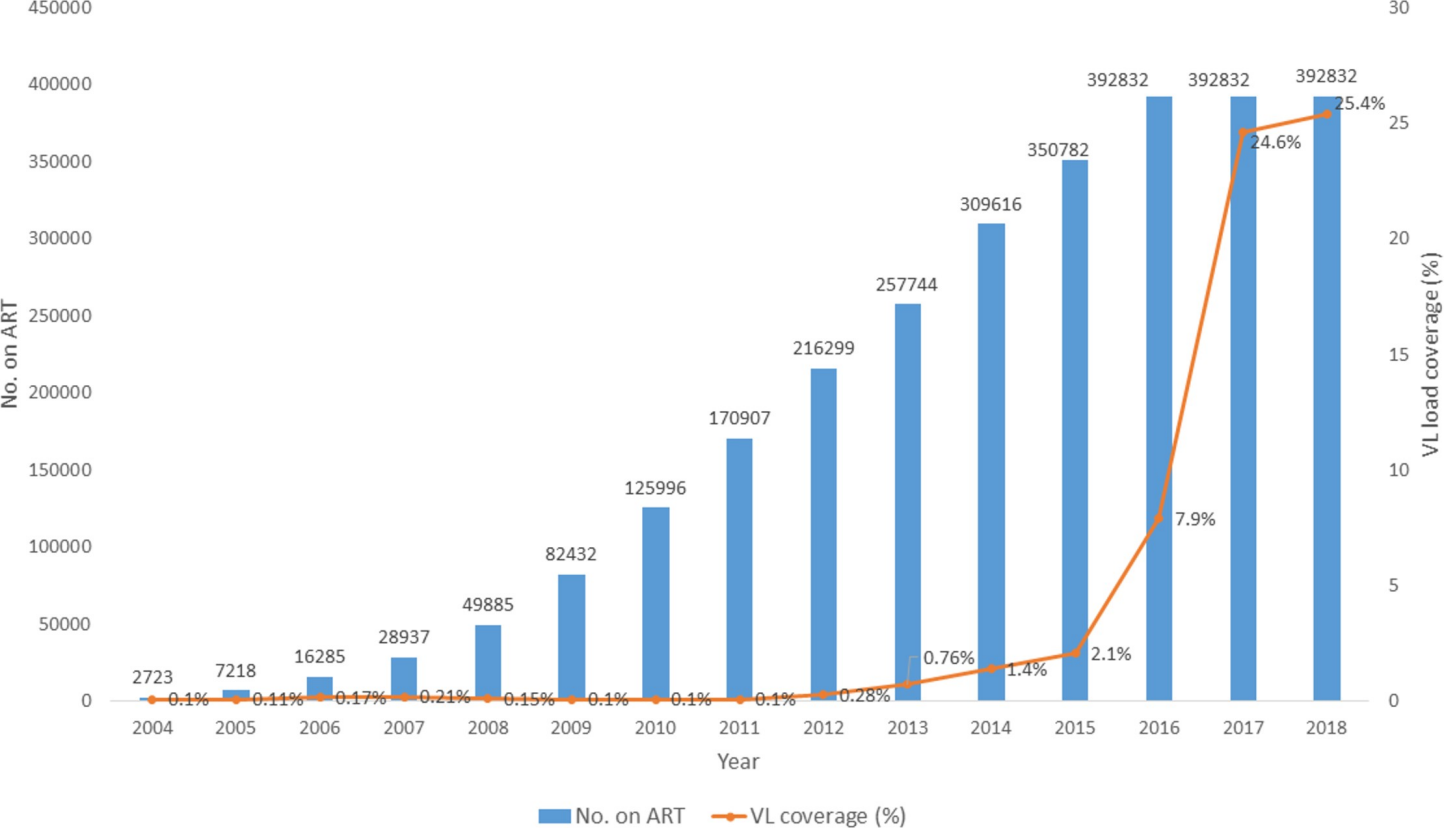
Trend in VL testing coverage and number of PLHIV enrolled in ART care between 2004 and 2017 in health facilities using ePMS with at least 12 months in ART care. PLHIV = people living with HIV; ART = antiretroviral therapy; VL = viral load; ePMS = electronic Patient Monitoring System. NB: *The numbers of patients enrolled in ART care are similar for 2016*,*2017 and 2018 as they refer only to those patients initiated on ART between 01 August 2004–01 January 2017 who were in ART care for at least 12 months and alive beyond 30 June 2017*. *The VL coverage extends to 2018 to account for patient started on ART from October-December 2016 onwards and eligible for a VL test after being in ART care for at least 12 months and also allowed for a window period for delayed access to a VL test*.

Overall, there were 392,832 study participants included in our study who had been initiated on ART and entered in the ePMS across 529 health facilities in Zimbabwe and had been on ART for at least 12 months. **Fig 2** shows the cumulative number of PLHIV initiated on ART by year versus the proportion with at least one VL test done, care for at least 12 months and entered in the ePMS. There was an exponential increase in VL coverage from <1% in 2004 when the public sector ART programme started to 25.4% by 2018 as the cumulative number increased from 2,723 to 392,832 over the same periods.

**Table 1** shows variations in the proportions of active patients on ART who had ever had a VL test by province, ranging from 43% in Manicaland to only 10% in Mashonaland Central. There were no gender differences observed (males: 24.9%; females: 25.7%). Higher proportions of adolescents (10–19 years) and patients aged above 50 years (29.05% and 29.28% respectively) had had a VL test compared with other age groups. Women who were not pregnant were more likely to have accessed at least one VL test compared to pregnant women (26.2% vs 17.5%). Those initiated on ART within the primary care level (22%) were less likely to have had a VL test than those at higher level facilities. Patients who had presented at ART enrollment with advanced HIV disease or with low CD4 of <200 cells per ml were more likely to have had at least one VL test done. Patients receiving 2^nd^ line ART were twice as likely to have had a VL test done (47.8%) compared to those on 1^st^ line ART (24.6%).

**Table 1:** Coverage of ever receiving viral load testing by demographic and clinical characteristics of PLHIV enrolled in the ART programme for >12 months in health facilities using the electronic Patient Management System between 2004 and 2017 in Zimbabwe.

Characteristic	Ever had a viral load test done?		
	Yes	No	P-value*
(N = 392,832)			
	N (%)	N (%)	
Total	99902 (25.43)	292930 (74.57)	
*Province (n = 392*,*831)*			
Harare	2467 (20.84)	9370 (79.16)	<0.001
Manicaland	19150 (42.63)	25776 (57.37)	
Mash Central	3332 (10.3)	29022 (89.7)	
Mash East	15982 (24.23)	49988 (75.77)	
Mash West	6949 (18.99)	29651 (81.01)	
Mat North	6332 (18.46)	27976 (81.54)	
Mat South	3698 (11.68)	27975 (88.32)	
Midlands	13364 (25.47)	39110 (74.53)	
Masvingo	16936 (36.44)	29537 (63.56)	
Bulawayo	11692 (32.28)	24524 (67.72)	
*Sex (n = 392*,*831)*			
Female	64431 (25.71)	186200 (74.29)	<0.001
Male	35471 (24.94)	106729 (75.06)	
*Age group at last visit*			
*(n = 392*,*822)*			
<10 yrs	2551 (23.16)	8462 (76.84)	<0.001
10–19 yrs	6453 (29.05)	15757 (70.95)	
20–29 yrs	8018 (19.48)	33150 (80.52)	
30–39 yrs	25723 (22.58)	88210 (77.42)	
40–49 yrs	32637 (27.03)	88121 (72.97)	
50+ yrs	24520 (29.28)	59220 (70.72)	
*Current pregnancy status at last visit (n = 250*,*630)*			
No	61840 (26.2)	173989 (73.8)	<0.001
Yes	2590 (17.5)	12211 (82.5)	
*Level of care at ART initiation (n = 392*,*831)*			
Primary health care	44682 (22.48)	154083 (77.52)	<0.001
First Referral Level	46041 (27.64)	120532 (72.36)	
Second Referral Level	5469 (36.35)	9575 (63.65)	
Third Referral Level	3710 (29.8)	8739 (70.2)	
*Baseline WHO Stage (n = 172*,*904)*			
I	7662 (22.82)	25917 (77.18)	<0.001
II	12932 (23.21)	42776 (76.79)	
III	19018 (23.72)	61162 (76.28)	
IV	1115 (32.44)	2322 (67.56)	
*Baseline CD4 count (cells/mL) (n = 115*,*158)*			
= /<200	15948 (30.4)	36511 (69.6)	<0.001
201–350	10223 (27.79)	26570 (72.21)	
351–500	3624 (23.57)	11751 (76.43)	
501/2000	2353 (22.34)	8178 (77.66)	
*Currently on TB treatment at last visit (n = 392*,*382)*			
Yes	1149 (27.9)	2973 (72.1)	<0.001
No	98753 (25.4)	289957 (74.6)	
*Anemia at baseline*			
*(n = 392*,*382)*			
Yes	744 (28.76)	1843 (71.24)	0.165
No	99158 (30.34)	291087 (69.66)	
*Current ART regimen at last visit (n = 391*,*300)*			
1st-line	92723 (24.64)	283587 (75.36)	0.222
2nd-line	7168 (47.82)	7822 (52.18)	

*The p-values shown are for the chi square test for associations between having a VL test ever done and the various demographic and clinical characteristics excluding unrecorded data for each variable.

**Fig 3:**
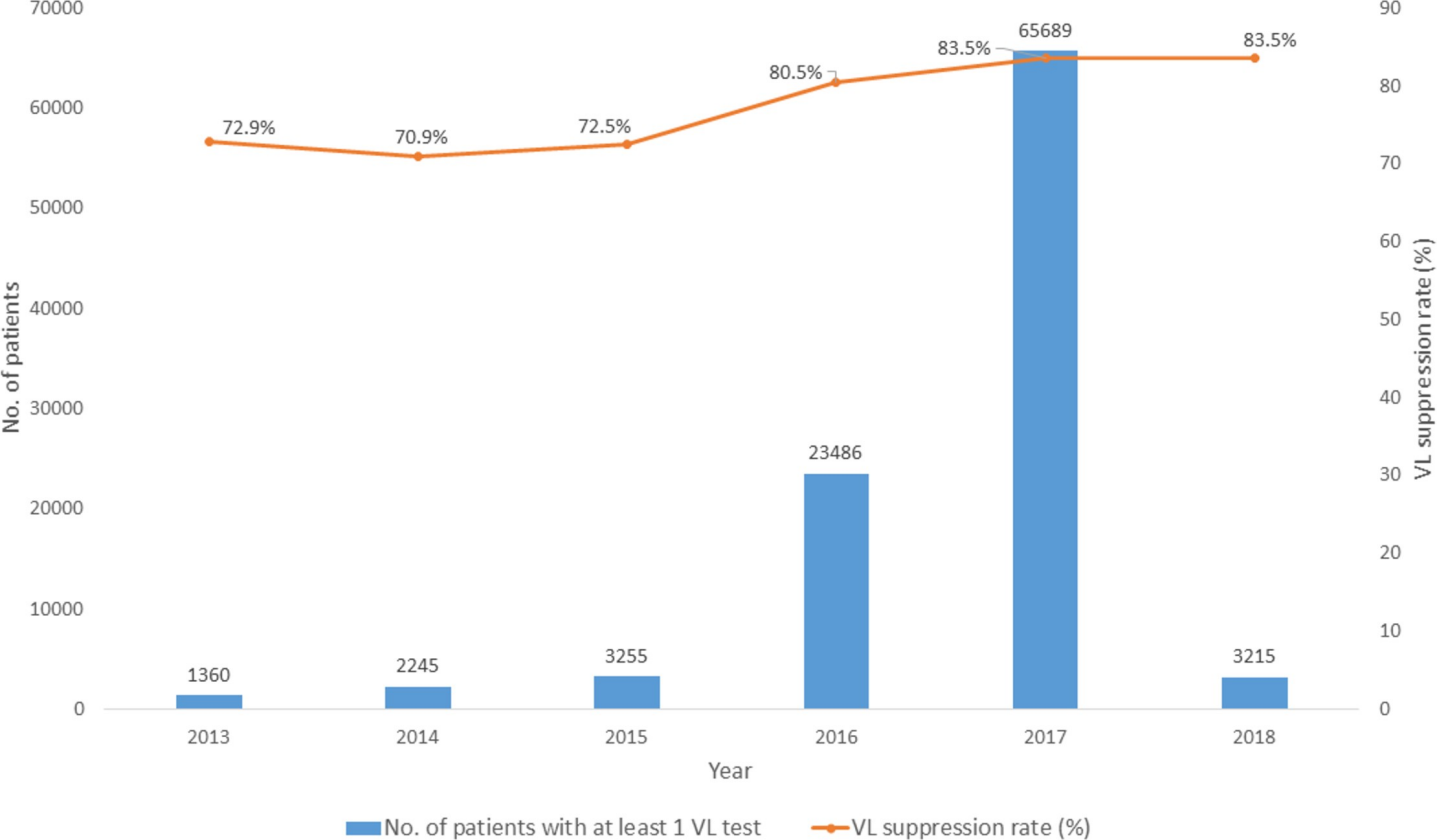
Number of PLHIV with at least one VL test done and proportions with viral suppression by year among those initiated ART between 2013–2017 and in care for at least 12 months at health facilities using ePMS and alive beyond 30 June 2017. VL = viral suppression; ART = antiretroviral therapy; ePMS = electronic Patient Monitoring System. *NB*: *652 patients with VL tests done between 2004 and 2012 out of the 99*,*902 with VL tests done between 2004 and 2018 have been excluded given the small numbers and the high probability of survival bias given that the ePMS was rolled out in January 2013 hence those who were alive among those initiated on ART before 2013 were likely entered in the system resulting in biased proportions with viral suppression*.

The proportion of PLHIV with viral suppression improved from 72.9% (95% confidence interval (CI): 70.4–75.2) in 2013 to 83.5% (95% CI: 83.2–83.8) in 2017(**Fig 3**).

**Fig 4:**
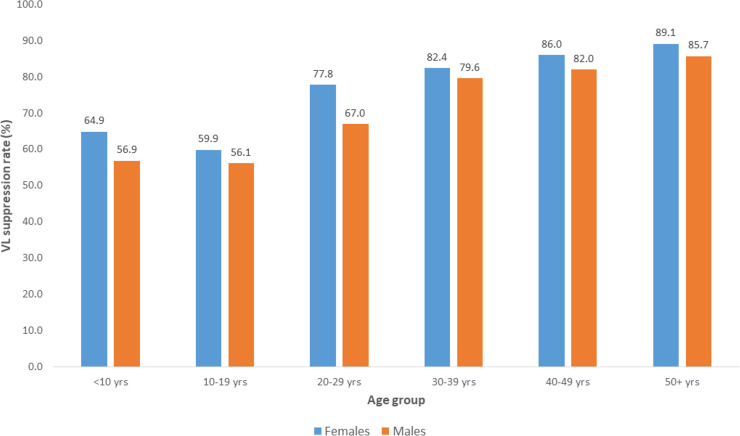
Comparison in proportions of PLHIV with VL suppression for the first VL test stratified by age-group and sex by year among those in ART care for at least 12 months between 2013 and 2017 at health facilities using ePMS.

**Fig 4** shows comparisons in proportions of PLHIV in ART care with suppressed VLs for the first VL test when stratified by age group and sex. Adolescents aged 10–19 years were least likely to be suppressed compared to other age groups. There were also significantly higher proportions with viral suppression among females compared to males for all pairwise comparisons by age group i.e. (p<0.001)

**Fig 5:**
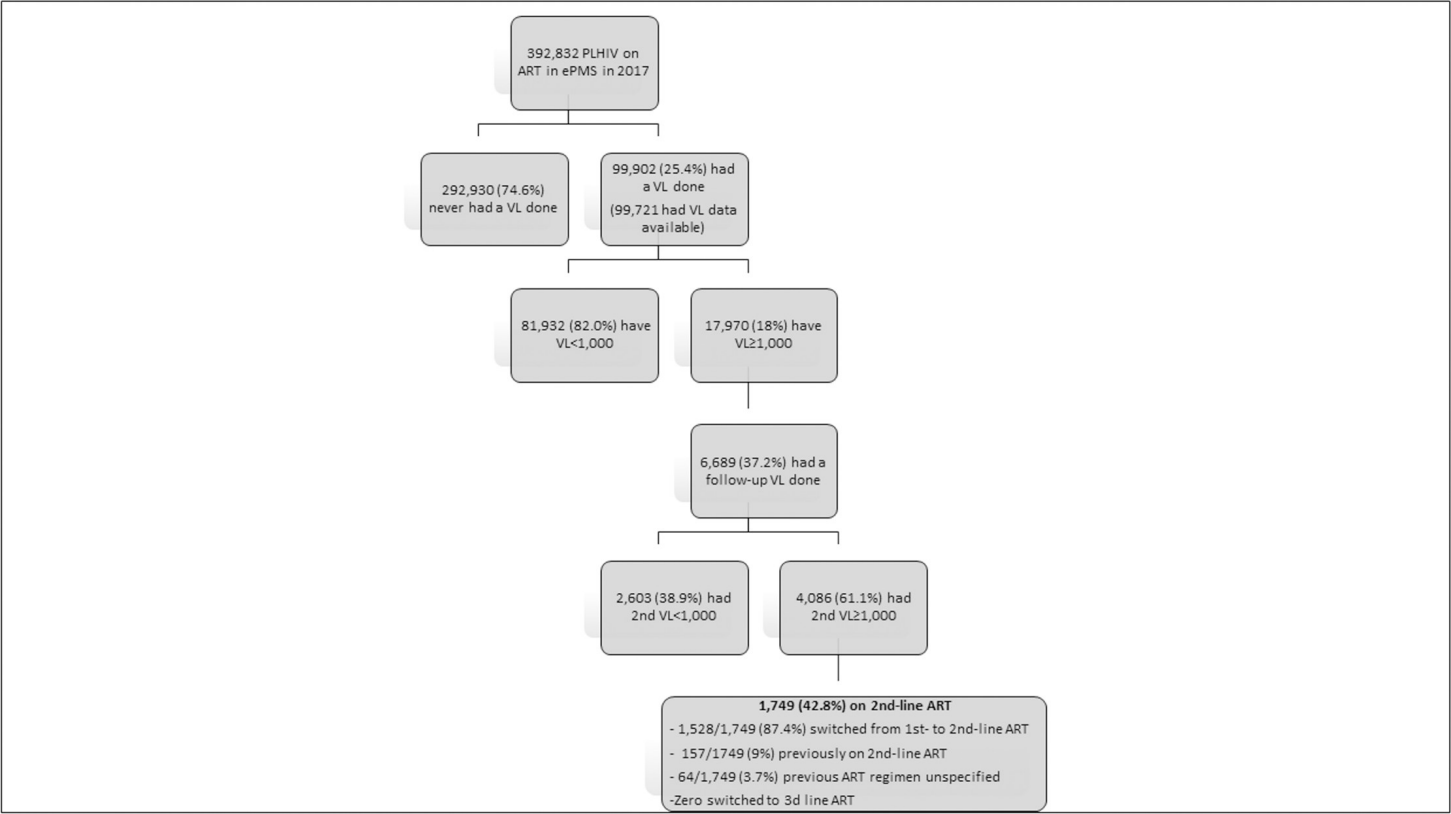
Flow chart showing the management of PLHIV still in ART care beyond 30 June 2017 and with unsuppressed viral load result in 2017 in the Zimbabwe ART program at health facilities using the electronic Patient Management System.

**Fig 5** shows a flow chart portraying uptake of an initial VL, proportion with unsuppressed VLs, subsequent access to follow-up VL test, confirmed virologic failure and proportions switched to second-line ARVs among PLHIV in ART care for at least 12 months and active in care beyond 31 March 2017. Of 392,832 PLHIV, 99,902 (25.4%) had ever had a VL test done. Median time to first VL test from start of ART was 63 months (IQR, 37–88) based on available data for 99,721 (99.8%) patients. Among the 99,721 active patients for whom VL data was available; 82% (81,932/99,721) were virally suppressed based on the initial VL test while the remainder 18% (17,970/99,721) had a VL of >1,000 copies/ml. Of the 17,970 with unsuppressed 1^st^ VLs, 37.2% (6,689/17,970) had a follow-up VL test. Data on dates for both 1^st^ and 2^nd^ VL tests was available for 6,314 /6,689 (94.4%) patients with 1^st^ VL test that was unsuppressed. Of these 6,314 patients, the time from the 1^st^ unsuppressed VL test to the 2^nd^ VL test was a median of 187 days, (IQR, 120–302).

Following an initial unsuppressed VL test result, among those who had a follow up VL test (n = 6689), 38.9% had a suppressed VL test at follow up. Fewer than half of the patients with a 2^nd^ unsuppressed VL test were switched to 2^nd^ line ART (42.8%; 1,749/4,086), of whom—1,528/1,749 (87.4%) switched from 1st- to 2nd-line ART whilst 157/1749 (9%) previously on 2nd-line ART and 64/1,749 (3.7%) had their ART regimen prior to the initial ART regimen unspecified. Of the 749 with complete data, time to switch to 2^nd^ line ART after the 2^nd^ VL test was a median of 89 days (IQR, 40–182).

A total of 17,970 active patients had an unsuppressed initial VL result (**Fig 5** and **Table 2**). Provinces with the highest proportions of patients with unsuppressed VL were Harare (21.1%), Mashonaland Central (21.2%) and Bulawayo (23%). Males compared to females had a higher proportion of patients with unsuppressed VL (21% vs 16.4%).

**Table 2:** Demographic and clinical characteristics of PLHIV on ART for ≥12 months with an initial unsuppressed viral load (>1000 copies/mL) at sites using the electronic Patient Management System from 2004 to 2017 in Zimbabwe.

Characteristic	Number with an initial VL test done	Number with an unsuppressed VL result	
		N	(%)
**Total**	**99,902**	**17,970**	**(17.99)**
*Province (n = 17*,*970)*			
Harare	2,467	520	(21.1)
Manicaland	19,150	3,692	(19.3)
Mash Central	3,332	707	(21.2)
Mash East	15,982	2,508	(15.7)
Mash West	6,949	1,167	(16.8)
Mat North	6,332	1,089	(17.2)
Mat South	3,698	720	(19.5)
Midlands	13,364	2,143	(16.0)
Masvingo	16,936	2,732	(16.1)
Bulawayo	11,692	2,692	(23.0)
*Sex (n = 17*,*970)*			
Female	64,431	10,539	(16.4)
Male	35,471	7,431	(21.0)
*Age group (n = 17*,*970)*			
<10 yrs	2,551	1,009	(39.6)
10–19 yrs	6,453	2,686	(41.6)
20–29 yrs	8,018	1,895	(23.6)
30–39 yrs	25,723	4,542	(17.7)
40–49 yrs	32,637	4,901	(15.0)
50+ yrs	24,520	2,937	(12.0)
*Current pregnancy status*			
*(n = 17*,*970)*			
No		10,121	(96.0)
Yes		418	(4.0)
*WHO staging (n = 7*,*315)*			
I	7,662	1,187	(15.5)
II	12,932	2,286	(17.7)
III	19,018	3,605	(19.0)
IV	1,115	237	(21.3)
*On TB treatment at enrolment (n = 17*,*970)*			
No	98,753	17792	(18.0)
Yes	1,149	178	(15.5)
*Baseline CD4 count (cells/mL) (n = 4*,*969)*			
= /<200	15,948	2,926	(18.4)
201–350	10,223	1,309	(12.8)
351–500	3,624	445	(12.3)
501/2000	2,353	289	(12.3)
*ART regimen prior to 1st VL test (n = 17*,*182)*			
1st-line	94,550	16,302	(17.2)
2nd-line	3,609	880	(24.4)
*Time to 1st VL test from ART initiation (n = 17*,*916)*			
≤6 months	1,610	493	(30.6)
7–12 months	4,319	743	(17.2)
1-2yrs	8,646	1,440	(16.7)
3-5yrs	32,754	5,968	(18.2)
>5yrs	52,392	9,272	(17.7)
*Level of Care (n = 17*,*970)*			
Primary health care	44,682	7400	(16.6)
First Referral Level	46,041	8412	(18.3)
Second Referral Level	5,469	1109	(20.3)
Third Referral Level	3,710	1049	(28.3)

PLHIV = People Living with HIV; ART = antiretroviral therapy; WHO = World Health Organization; TB = tuberculosis; VL = viral load.

: Patients have been in active in care for ≥12 months beyond 30 June 2017.

**Table 3** below shows that the prevalance of virologic failure varies by province and age with younger age groups particularly the adolescents (ARR 1.34; 95%CI: 1.25–1.44) and pediatrics (ARR 1.34; 95% CI: 1.22–1.46) being more likely to have virologic failure. Patients on second line ART were 30% less likely to have virologic failure on a repeat VL test compared to those receiving first line ART (ARR 0.70; 95% CI: 0.62–0.78). Time taken to initial VL test was an important predictor of virologic failure in repeat testing. Patients who had been on ART for >5 years before having their first VL test were 46% more likely to have virologic failure (ARR 1.46; 95% CI: 1.24–1.71) compared to those who had the initial VL ≤6 months post ART initiation.

**Table 3:** Generalized linear regression model of demographic & clinical characteristics associated with confirmed virologic failure on repeat testing among PLHIV in ART care for ≥12 months at health facilities using the electronic Patient Management System from 2004 to 2017 in Zimbabwe.

Characteristic	Total	Repeat VL>1000 copies/ml	RR (95% CI)	ARR (95% CI)
	N	n (%)		
**Total**	**6689**	**4086 (61.1)**		
*Province*				
Harare	313	151 (47.9)	Reference	Reference
Manicaland	1934	1254 (64.9)	1.35 (1.20–1.53)	1.61 (1.37–1.89)
Mash Central	107	81 (75.0)	1.56 (1.34–1.83)	1.83 (1.52–2.21)
Mash East	798	468 (58.7)	1.22 (1.08–1.39)	1.51 (1.28–1.79)
Mash West	371	194 (52.2)	1.09 (0.94–1.26)	1.38 (1.15–1.66)
Mat North	266	138 (51.9)	1.08 (0.92–1.27)	1.28 (1.05–1.55)
Mat South	108	79 (73.2)	1.53 (1.30–1.79)	1.82 (1.49–2.22)
Midlands	637	489 (76.9)	1.60 (1.42–1.81)	1.85 (1.57–2.18)
Masvingo	976	567 (58.1)	1.21 (1.07–1.38)	1.47 (1.25–1.74)
Bulawayo	1179	665 (56.5)	1.18 (1.04–1.34)	1.35 (1.17–1.56)
*Sex*				
Female	3,919	2369 (60.5)	Reference	Reference
Male	2,770	1717 (62.0)	1.03 (0.99–1.07)	1.03 (0.99–1.07)
*Age group*				
50+ yrs	1,173	616 (52.5)	Reference	Reference
40–49 yrs	1,789	1055 (56.0)	1.12 (1.05–1.20)	1.12 (1.05–1.20)
30–39 yrs	1,500	922 (61.5)	1.17 (1.09–1.25)	1.19 (1.11–1.28)
20–29 yrs	629	409 (65.0)	1.24 (1.14–1.34)	1.27 (1.18–1.38)
10–19 yrs	1,186	811 (68.4)	1.30 (1.22–1.39)	1.34 (1.25–1.44)
<10 yrs	412	273 (66.3)	1.26 (1.16–1.38)	1.34 (1.22–1.46)
Current pregnancy status				
No	3,792	1495 (39.4)	Reference	Reference
Yes	127	55 (43.3)	0.94 (0.80–1.09)	1.01 (0.82–1.25)
*WHO Stage*				
I	381	158 (41.5)	Reference	Reference
II	820	304 (37.1)	1.08 (0.98–1.20)	1.04 (0.95–1.15)
III	1,338	526 (39.3)	1.04 (0.94–1.14)	1.01 (0.92–1.11)
IV	101	41 (40.6)	1.02 (0.85–1.22)	0.98 (0.83–1.17)
Not recorded	4,049	1574 (38.9)	1.05 (0.96–1.15)	1.05 (0.96–1.15)
*On TB treatment at enrolment*				
No	6,614	2297 (60.6)	Reference	Reference
Yes	75	72 (56.7)	1.03 (0.86–1.22)	-
*Baseline CD4 count (cells/mL)*				
= /<200	1,075	677 (63.0)	Reference	Reference
201–350	446	243 (54.5)	0.87 (0.79–0.95)	0.85 (0.78–0.94)
351–500	123	63 (51.2)	0.81 (0.68–0.97)	0.83 (0.70–0.98)
>500	80	34 (42.5)	0.67 (0.52–0.87)	0.70 (0.54–0.89)
Not recorded	4,965	3069 (61.8)	0.98 (0.93–1.03)	0.94 (0.89–0.99)
*ART regimen prior to 1st VL test*				
1st-line	6,055	3805 (62.8)	Reference	Reference
2nd-line	356	160 (44.9)	0.72 (0.64–0.8)	0.70 (0.62–0.78)
Not recorded	278	121 (43.5)	0.69 (0.6–0.79)	0.76 (0.66–0.88)
*Time to 1st VL since ART initiation*				
≤6 months	238	97 (40.8)	Reference	Reference
7–12 months	280	141 (50.4)	1.24 (1.02–1.5)	1.16 (0.96–1.41)
1-2yrs	510	296 (58.0)	1.42 (1.2–1.69)	1.32 (1.12–1.57)
3-5yrs	2,269	1373 (60.5)	1.48 (1.27–1.74)	1.37 (1.17–1.61)
>5yrs	3,369	2165 (64.3)	1.58 (1.35–1.84)	1.46 (1.24–1.71)
Not recorded	23	14 (60.9)	1.49 (1.04–2.14)	1.59 (1.10–2.29)
*Level of Care*				
Primary health care	2,221	1411 (63.5)	Reference	Reference
First Referral Level	3,274	1930 (59.0)	0.93 (0.89–0.97)	0.90 (0.86–0.94)
Second Referral Level	521	379 (72.7)	1.15 (1.08–1.22)	1.00 (0.94–1.06)
Third Referral Level	673	366 (54.4)	0.86 (0.79–0.92)	1.03 (0.93–1.15)

VL = viral load (copies/mL); HIV = Human Immunodeficiency Virus; ART = antiretroviral therapy; IQR = inter-quartile range; WHO = World Health Organisation; TB = Tuberculosis; ART = antiretroviral therapy; RR = relative risk; ARR = multivariate-adjusted relative risk; CI = confidence interval.

## Discussion

This is the first study to assess provision of public sector VL testing services in Zimbabwe using the ePMS system, demonstrating its ability to track patients in general and VL testing scale up overtime. The study results revealed that only a quarter (25.4%) of the active ART patients had ever had a VL test done, and among those with a first high VL, 37.2% had a repeat VL test done. As shown in population based and clinical studies; high-risk populations included adolescents and males who were least likely to have an initial suppressed VL [11, 12]. Proportions of PLHIV with VL suppression were high and had improved from 72.9% in 2013 to over 83.5% in 2018 similar to what has been reported elsewhere [7]. However, the proportions with VL suppression were lower than what has been reported in other African countries such as Botswana (95.6%), Malawi (90.8%) and Rwanda (93.2%) [12].

This study used secondary analysis of data collected via Zimbabwe’s ePMS deployed at most high-volume health facilities providing ART services in Zimbabwe, across all the four tiers of the health care delivery system and includes 840,971 PLHIV. Of note 75% of all ART patients in Zimbabwe are captured within ePMS (the system excludes low volume health facilities including some private sector health facilities).

Importantly only a quarter of the active ART patients had accessed an initial VL test by end 2017 which is in contrast to higher VL testing coverage of over 75% of individuals on ART each year reported in recent years in some African countries including South Africa, Namibia, Kenya, and Uganda [13]. Other countries such as Tanzania reported low VL coverage at 9% [14]. This calls for urgent attention to treatment monitoring and especially among patients decentralized to primary care facilities who had less access to a VL test. A systematic review conducted by Pham et al (2017) revealed low coverage of monitoring tests in rural areas [15]. Improved coverage of VL testing would enable clinicians to determine which patients are stable on treatment, and eligible for reduced annual clinical reviews as outlined in Zimbabwe’s differentiated care policy for stable patients [16, 17].

Possible reasons for the low VL coverage include limited VL testing capacity; inadequate and centralized VL platforms with limited Point of Care VL devices, limited transportation systems of specimens from peripheral health facilities to central laboratories, stock-outs of reagents; and few laboratory scientists to perform the tests. A survey conducted by WHO in low to middle-income countries (LMICs) in 2014 cited several reasons for slow implementation of VL testing including financial constraints, insufficient and overburdened healthcare professionals, poor training and lack of knowledge, and weak transport and laboratory systems [18]. The national ART program is reviewing its implementation bottlenecks for the VL strategy with a view to fast track its national rollout. The recent introduction of Point of Care VL technologies in Zimbabwe for remote areas presents an opportunity to address equity concerns for VL access. Patient education on the benefits of VL testing would likely create demand for the service.

The median time from ART enrolment to 1^st^ VL test was over 5 years, because scale up of routine VL testing only started in 2016. We would anticipate that time to first VL will reduce as roll out continues. Health care worker training will be critical to ensure timely testing and that results are acted on appropriately.

As shown, management of patients with an unsuppressed initial VL test was sub-optimal. Around a third (37%) had a repeat VL test done within 9 month of the first test, despite guidelines for 100% retesting by 3 months. Wide variations in the uptake of a repeat VL test following an unsuppressed VL exists in literature. A study in Mozambique during 2014–2015 at MSF facilities showed comparable low proportions of patients with follow-up VL tests done among those with a high first VL result (35%) [19] with 88.5% reported from a rural district in Rwanda [12]. National ART Guidelines state that patients with an initial unsuppressed VL results should undergo 3 EAC sessions followed by a repeat VL test after 3 months to confirm viral failure. Possible reasons for low uptake of the repeat VL test may be limited awareness of the VL testing algorithm and poor documentation of the VL test result.

Of concern were delays in switching patients to 2^nd^ line ART after the 2^nd^ VL test (median of 89 days; IQR, 40–182) which contrasts other studies in rural Rwanda (median of 17 days IQR: 8–42)) [12]. Lengthy delays in switching patients to second line ART have also been reported in KwaZulu, Natal, South Africa (median time of 6.4 months; IQR 0–43.3 months) and in Uganda (8.1 months; IQR: 3.7–17) [20, 21]. The VL sample transport system and feedback of the results has been inefficient and fragmented with multiple courier systems, lack of monitoring tools and, as a result, limited accountability between the couriers at peripheral and FEDEX at the collection sites. This has resulted in long turn- around times from VL sample collection to feedback of the VL results. Frequent downtimes laboratory equipment have also contributed to the long VL result turn-around times, all these contributing to delays in switching patients with unsuppressed VLs to 2^nd^ line ART.

Fewer than half of active patients (42.8%) with a 2^nd^ unsuppressed VL test were switched to a 2^nd^ line ART regimen, suggesting that dissemination and training of clinicians in the revised ARV Guidelines and the management of treatment failure has not been effective. A systematic review from 16 sub-Saharan countries showed that 58% of patients with confirmed virologic failure were switched to second line ART [22]. Clearly timely identification and management of treatment failure cases to avert emergence of opportunistic infections and mortality from high- risk patients are crucial to the ART program.

The levels of viral suppression among patients enrolled into the national ART program falls below the UNAIDS 90-90-90 fast track targets. No gender differences were observed in accessing VL testing although in other studies [23] more women than men accessed the test. Despite equal access to VL testing; males tended to experience viral failure more commonly than females and similar findings were observed in the Zimbabwe Population-based HIV Impact Assessment (ZIMPHIA) [7]. A previous study in Zimbabwe showed that males had higher patient attrition and mortality compared to females due partly to late presentation for HIV treatment and care [24]. A case-control study conducted in Burkina Faso by Penot P. et al in 2012, showed a strong association between male gender and virologic failure [25].

In 2019 Zimbabwe introduced dolutegravir, a potent integrase inhibitor as part of its first line therapy. It has a higher barrier to resistance than efavirenz and fewer side effects. It is hoped that this change in regimen will support a higher proportion of patients achieving viral suppression.

Children and adolescent groups were the least likely to be virally suppressed (VL<1,000 copies/ml), consistent with the ZIMPHIA survey findings and studies conducted in Uganda, Swaziland, Kenya and Mozambique [7, 23, 26, 27]. Poor adherence to ARV drugs is common in children and adolescents. In children this is due to inadequate medication formulations, difficulties in administering medicines, drug toxicities and side effects, social context (example lack of consistent caregiver). Adolescents frequently find long-term medication adherence difficult including HIV treatment [28] while some of them are not fully disclosed to of their HIV status impacting negatively on their perceptions of, and importance of life-long HIV treatment. The national program should prioritize interventions targeted at children and adolescents including implementing differentiated service delivery models for adolescents such as provision of adolescent-friendly services, peer- to- peer psychosocial support and counselling services as well as encouraging adolescents to use reminders to take their pills. Health workers should work closely with caregivers of infected children to provide adherence assessment, support, and education.

Lastly, factors associated with virologic failure included patients receiving 1st line ART and patients with longer duration on ART. A recent study [29] conducted in Harare City provided a similar findings where those on 2nd line ART (when compared to being on 1st line ART) were more likely to have viral suppression, among patients with an initial unsuppressed VL. The initial unsuppressed VL could be due to poor initial adherence, which was later corrected by enrolment into EAC and the use of superior 2^nd^ line Protease Inhibitor medicines. We also found in our study that children and adolescents were more likely to have virologic failure and this has been observed elsewhere, including people with low recent CD4 below 350 [27]. This is likely due to psychosocial issues faced by these age groups (particularly the adolescents) whereby they remain non-adherent to life-long therapy despite underoing EAC sessions.

This study has several strengths including: use of a huge data set with 75% of all ART patients in Zimbabwe assessed over 13 years of follow up; the assessment of recent (2017) VL scale up implementation and providing opportunities for quality improvement projects.

Study limitations included the exclusion of health facilities that had not started using the ePMS. These are typically primary care facilities with low client volumes in rural settings. Another limitation was that we exclusively used and analyzed programme data and so were restricted to analysis of pre-determined data variables with some incomplete and inaccurate data entries. Critically we were unable to assess whether patients received EAC sessions following an initial unsuppressed VL test result or the socio-economic and educational status of patients. We were also unable to ascertain whether a VL test was conducted based on clinical need (ie ‘targeted’ testing) or as part of ‘routine’ scale up. Finally, we might have under-estimated the uptake of VL testing services where documentation of medical records is poor, as our study relied on a documented VL test result.

In conclusion, proportions of PLHIV with VL suppression improved from 72.9% in 2013 to over 83.5% in 2018. The government’s policy of using VL testing as a routine practice for monitoring adherence and treatment response provided opportunities to improve patient monitoring and treatment outcomes however, due to its implementation gaps, treatment was not fully optimized. Strengthening the capacity for VL testing, the identification of high- risk patients with viral failure and prompt utilization of the test results is paramount for treatment optimization. A quality improvement (QI) intervention has been planned in Zimbabwe to respond to these suboptimal results. The ePMS has demonstrated its usefulness in assessing the performance of the national VL testing program albeit some limitations, in order to help policy- makers to address identified bottlenecks that impede progress.
